# iNOS in Macrophage Polarization: Pharmacological and Regulatory Insights

**DOI:** 10.3390/ijms262412056

**Published:** 2025-12-15

**Authors:** Ji-Hye Kang, Ji-Hee Kim, Jeong-An Gim, Mi-Young Lee

**Affiliations:** 1 Department of Medical Biotechnology, College of Medical Science, Soonchunhyang University, Asan 31538, Chungnam, Republic of Korea; 2 Department of Occupational Therapy, College of Medical Science, Soonchunhyang University, Asan 31538, Chungnam, Republic of Korea; 3 Institute for Molecular Metabolism Innovation, Soonchunhyang University, Asan 31538, Chungnam, Republic of Korea; 4 Department of Medical Science, College of Medical Science, Soonchunhyang University, Asan 31538, Chungnam, Republic of Korea

**Keywords:** inducible nitric oxide synthase (iNOS), macrophage polarization, synthetic iNOS regulator, natural iNOS regulator, clinical translation of iNOS inhibitor

## Abstract

Macrophages play a central role in orchestrating inflammation and immune responses, with their polarization states (M1 or M2) exhibiting both pro- and anti-inflammatory, as well as anti-cancer functions. Inducible nitric oxide synthase (iNOS) plays a pivotal role in immune regulation through its impact on macrophage polarization. As a hallmark of M1 macrophages, iNOS drives pro-inflammatory responses, while its suppression favors anti-inflammatory M2 phenotypes. This dual role as both a biomarker and regulatory hub positions iNOS as a promising therapeutic target across diverse disease states. This review examines the roles of iNOS modulation in macrophage polarization across various disease models. Both synthetic and natural iNOS regulators have been shown to modulate iNOS activity, thereby reprogramming macrophages toward either inflammatory or reparative phenotypes. We categorize these regulators into those that directly inhibit enzymatic activity and those that indirectly modulate through signaling pathways. Additionally, recent preclinical and clinical studies on iNOS inhibitors are summarized, highlighting their therapeutic potential in inflammatory, metabolic, and oncologic diseases. Taken together, the evidence underscores the therapeutic relevance of iNOS regulation as a strategy to reprogram macrophage plasticity in disease-specific immune microenvironments.

## 1. Introduction

Nitric oxide (NO) is a versatile and multifunctional signaling molecule involved in numerous physiological and pathological processes. It is synthesized by the nitric oxide synthase (NOS) enzyme family, which includes inducible NOS (iNOS/NOS2), endothelial NOS (eNOS/NOS3), and neuronal NOS (nNOS/NOS1). Three NOS isoforms catalyze the same chemical reaction, L-arginine to L-citrulline + NO, whereas their differential structural domains, regulatory mechanisms, and subcellular localizations allow for finely modulated control of nitric oxide production across diverse physiological contexts [1].

Although inducible (iNOS), endothelial (eNOS), and neuronal (nNOS) nitric oxide synthases share a conserved modular architecture—including an N-terminal oxygenase domain and a C-terminal reductase domain connected via a calmodulin-binding linker—the three isoforms exhibit distinct structural and regulatory features that define their isoform-specific functions [2].

Notably, inducible nitric oxide synthase (iNOS) is transcriptionally upregulated in response to inflammatory stimuli and generates high-output nitric oxide (NO) in a calcium-independent manner, owing to its strong constitutive binding to calmodulin (CaM). In contrast, endothelial (eNOS) and neuronal (nNOS) isoforms are calcium/CaM-dependent enzymes that remain inactive under basal conditions and are transiently activated upon specific signaling events.

At the catalytic site level, subtle amino acid variations in the substrate access channel, tetrahydrobiopterin (BH4) binding pocket, and adjacent loops contribute to differences in enzymatic efficiency and inhibitor sensitivity. These structural divergences have been exploited in the design of isoform-selective NOS inhibitors, particularly those that selectively target the iNOS heme- or BH_4_-binding environment [3].

iNOS is primarily induced in immune cells such as macrophages and T lymphocytes upon exposure to pro-inflammatory signals including lipopolysaccharide (LPS), interleukin-1β (IL-1β), tumor necrosis factor-α (TNF-α), and interferon-γ (IFN-γ) [4]. These stimuli activate transcription factors such as NF-κB and STAT-1α, which bind to the iNOS promoter and initiate gene transcription [5].

While iNOS is generally inducible in immune contexts, notably, it is constitutively active in the healthy human paranasal epithelium, producing high levels of nitric oxide (NO) that contribute to mucosal immunity [6]. In support of its protective role, reduced nasal NO levels have been observed in patients with chronic rhinosinusitis, and pharmacological inhibition of nasal iNOS has been shown to precipitate acute sinusitis [7]. In tuberculosis, iNOS limits neutrophil recruitment and pathogen proliferation, exerting anti-inflammatory effects [8].

As iNOS is involved in both promoting and resolving inflammation, recent therapeutic strategies targeting iNOS, including selective small molecule inhibitors and natural regulators, have demonstrated promising results in in vitro and in vivo models. These approaches aim to reduce NO-mediated cytotoxicity, restore mitochondrial integrity, and enhance immune responses. However, clinical translation remains limited due to challenges in specificity, delivery, and potential immunosuppressive effects. Immunotherapeutic strategies have demonstrated the capacity to reprogram immune cell function, including macrophage polarization via iNOS-related mechanisms. Macrophages respond dynamically to environmental cues and are broadly categorized into classically activated (M1-like, pro-inflammatory) or alternatively activated (M2-like, anti-inflammatory) phenotypes. M1-like macrophages are characterized by high iNOS expression and nitric oxide (NO) production, supporting antimicrobial, pro-inflammatory, and tumoricidal functions. In contrast, M2-like macrophages exhibit low iNOS levels and promote anti-inflammatory, tissue remodeling, angiogenesis, and immune regulation. iNOS thus functions not only as a critical enzyme in M1 macrophages but also as a molecular marker distinguishing inflammatory from regulatory macrophage states [9].

The existing literature includes several reviews that address the role of macrophage reprogramming in disease modulation, particularly in cancer and chronic inflammation.

For instance, Kashfi et al. provided a broad overview of macrophage phenotypes in cancer therapy, highlighting the reprogramming of tumor-associated macrophages (TAMs) as a promising therapeutic avenue [10]. Similarly, Rannikko et al. summarized the current clinical landscape of macrophage-targeting strategies, focusing on TAM-directed therapeutics but without emphasis on specific enzymatic targets such as iNOS [11]. Furthermore, Ekmekcioglu et al. explored the potential of iNOS inhibition to improve the efficacy of cancer immunotherapies, although their discussion centers more on iNOS in the context of immune checkpoint modulation than on macrophage polarization by itself [12].

This review highlights inducible nitric oxide synthase (iNOS) as both a biomarker and a pivotal regulatory node in macrophage polarization, offering a comparative evaluation of direct and indirect iNOS inhibitors—including synthetic compounds and natural products.

By elucidating the context-dependent effects of these agents across diverse immunological milieus, from inflammatory disorders to the tumor microenvironment, this work proposes a strategic framework for leveraging iNOS modulation in disease-specific macrophage reprogramming and immune intervention.

## 2. Biological Concepts of iNOS Modulation and Macrophage Polarization for Therapeutic Strategy

### 2.1. Structure and Function of iNOS

iNOS is a homodimeric enzyme composed of two identical subunits, each containing a modular domain architecture that supports the enzymatic production of nitric oxide (NO) from L-arginine [13].

Each NOS monomer comprises an N-terminal heme-containing oxygenase domain and a C-terminal flavin-containing reductase domain, connected by a calmodulin-binding region essential for electron transfer. The oxygenase domain houses the active site for L-arginine binding, the heme iron for oxygen catalysis, and the cofactor BH4 [14,15].

The reductase domain, containing flavin adenine dinucleotide (FAD) and flavin mononucleotide (FMN), mediates electron transfer from NADPH to the heme center via a stepwise pathway (NADPH → FAD → FMN → heme) to fuel the hydroxylation of L-arginine to form NO and L-citrulline [14,15].

Because iNOS functions not only as a key biomarker of macrophage activation but also as a regulatory hub in immune signaling, it has been recognized as a promising therapeutic target in a range of pathological conditions—including chronic inflammation, autoimmune disease, atherosclerosis, neurodegeneration, and cancer [16].

### 2.2. iNOS Signaling Pathways

**Figure 1:**
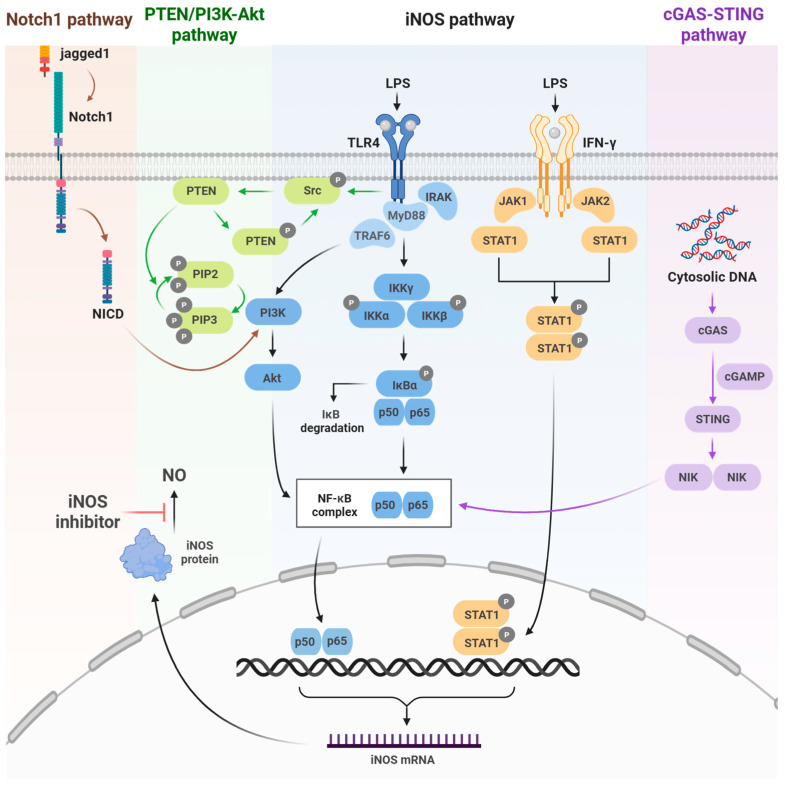
Inducible nitric oxide synthetase (iNOS) signaling pathways. Schematic representation of the key upstream pathways regulating iNOS activation, including Notch-1, PTEN/PI3K-Akt, and cGAS–STING signaling, which activate NF-κB to induce NOS2 expression. Color-coded arrows indicate each pathway. Created in BioRender. Kang, J. (2025) https://BioRender.com/wj2ctnp (accessed on 8 December 2025).

iNOS expression is governed not by a single pathway, but by a complex regulatory network in which diverse inflammatory receptors and immune stimuli initiate NOS2 transcription through distinct mechanisms. Representative upstream regulatory pathways include the Notch1, PTEN/PI3K–Akt, TLR4/NF-κB, JAK/STAT1, and cGAS–STING signaling (Figure 1).

The TLR4/NF-κB pathway is activated by LPS stimulation. Subsequent IKK activation leads to the phosphorylation and degradation of IκB, allowing NF-κB (p50/p65) to translocate to the nucleus and promote iNOS expression. The JAK/STAT1 pathway similarly promotes iNOS expression via STAT1 activation downstream of IFN-γ receptor signaling. These two pathways are central regulators of NOS2 transcription [17]. Notch1 signaling promotes iNOS transcription by activating NF-κB through Akt signaling, which is triggered by NICD generation following ligand (Jagged1) binding [18].

The PTEN/PI3K–Akt pathway modulates NF-κB activity through PI3K activation, Akt phosphorylation, and suppression of PTEN enhances PI3K/Akt–NF-κB signaling, thereby increasing iNOS expression [19].

Finally, the cGAS–STING pathway activates the cGAMP–STING signaling cascade by cytoplasmic DNA, which activates NF-κB and IRF3 [20]. Collectively, these upstream signaling pathways induce NOS2 transcription by activating key transcription factors such as NF-κB and STAT1, thereby regulating iNOS expression.

iNOS activity generates NO from L-arginine and contributes to downstream processes that influence macrophage polarization. NO reacts with superoxide to form peroxynitrite (ONOO^−^) [21]. NO and peroxynitrite are reactive nitrogen species (RNS) that function not only as toxic molecules but also as intracellular signaling molecules [22]. Such redox dynamics are known to simultaneously regulate transcription factors, including NF-κB, HIF-1α, and Nrf2, which also exhibit functional crosstalk [23]. Changes in the activity of these transcription factors act as a key mechanism for determining the M1/M2 polarization states by reprogramming inflammatory gene expression and cellular metabolism [24].

Furthermore, NO influences mitochondrial respiration. Many studies have reported that NO and peroxynitrite suppress or impair the activities of respiratory enzyme complexes I, II, III, and IV, resulting in the inhibition and loss of mitochondrial electron transport chain (ETC) components [25,26]. NO also inhibits pyruvate dehydrogenase (PDH), thereby limiting mitochondrial oxidative metabolism, enhancing glycolysis, and reinforcing M1 polarization in macrophages. Additionally, NO-dependent PDH inhibition reduces pyruvate conversion to acetyl-CoA, reduces TCA cycle flux, and further reinforces glycolytic reprogramming during M1 polarization [27,28].

Crosstalk between the iNOS signaling pathway and macrophage polarization involves the mTOR downstream effectors of PI3K/Akt signaling [29]. Activation of mTOR complex 1 (mTORC1) enhances glycolysis and promotes M1 polarization, whereas activation of mTOR complex 2 (mTORC2) increases oxidative metabolism and supports M2 polarization.

In addition, peroxynitrite generated by NO induces DNA strand breaks in both nuclear and mitochondrial DNA [30,31]. This DNA damage may contribute to the reactivation of the cGAS–STING pathway, forming a potential positive feedback loop in macrophages [32].

Nitric oxide production plays a key role in the pathogenesis of various diseases, including stroke, myocardial infarction, chronic heart failure, diabetes, circulatory shock, chronic inflammatory diseases, cancer, and neurodegenerative disorders [33]. In the acute phase, iNOS induction generates high levels of NO and superoxide, forming peroxynitrite. This results in rapid mitochondrial inhibition, DNA damage, PARP activation, and acute tissue injury, such as in ischemia–reperfusion injury and acute inflammation. In the chronic phase, prolonged nitrative stress has been reported to promote DNA mutations (8-nitroguanine formation), inhibit DNA repair, activate matrix metalloproteinases (MMPs), and drive fibrosis and tissue remodeling, thereby exacerbating cancer development, chronic heart failure, and the progression of chronic inflammatory diseases.

Collectively, these downstream metabolic changes and signaling crosstalk, together with upstream regulators of iNOS, constitute key mechanisms that govern macrophage phenotype and shape pathological outcomes.

### 2.3. iNOS Modulation Strategies: Direct and Indirect Approaches

iNOS is a key enzyme that contributes to the pathological processes in a wide range of inflammatory, autoimmune, and neurodegenerative diseases. Therefore, targeting iNOS has become a promising strategy in drug development. Two broad approaches exist: direct iNOS inhibitors that bind to the enzyme and block its activity, and indirect inhibitors that modulate upstream pathways controlling iNOS expression.

Direct iNOS inhibitors, mainly synthetic chemicals such as 1400 W, L-NIL, GW274150, 2-Iminobiotin, interact directly with the enzyme’s active site or its cofactors such as heme or BH4, leading to immediate and selective inhibition of NO production [34,35,36].

In contrast, indirect iNOS inhibitors typically consist of multi-target natural products or immunomodulatory agents, such as dexamethasone, which exert their effects through upstream signaling pathways rather than direct enzyme inhibition [37].

Indirect inhibitors suppress NOS2 gene transcription and iNOS activity by regulating signaling pathways and transcription factors like NF-κB, STAT1, or by reducing upstream inflammatory mediators such as cytokines and reactive oxygen species (ROS). Thus, direct inhibitors act rapidly with high isoform-selectivity, whereas indirect inhibitors act more slowly and affect multiple inflammatory pathways with broader selectivity. In addition, it is possible that direct inhibitors may have lower off-target toxicity, whereas indirect inhibitors may exert systemic effects due to extensive pathway cross-talk [34,38].

Several pieces of evidence support the concept that direct iNOS inhibitors are especially advantageous in acute injury models, such as traumatic brain injury or acute ischemic stroke, where rapid suppression of NO production mitigates secondary damage [35].

On the other hand, indirect inhibitors demonstrate efficacy in chronic inflammatory conditions (e.g., arthritis, joint disorders), where modulation across multiple signaling pathways and long-term immune regulation are required [39].

### 2.4. Role of iNOS in Macrophage Polarization

iNOS serves not only as a hallmark enzyme of M1 polarization but also as a functional driver of immune responses. Its activity is tightly regulated by inflammatory stimuli, including IFN-γ, LPS, and TNF-α, which act via NF-κB and STAT1 signaling pathways. In contrast, M2 polarization is favored by IL-4/IL-13 signaling through STAT6, which downregulates iNOS while promoting Arg1 expression. These opposing programs form the basis of a finely balanced immune system, where perturbations in iNOS expression can drive macrophage fate toward either inflammatory or immunoregulatory phenotypes.

Inhibiting or reprogramming iNOS-driven macrophage responses offers a strategy to either dampen pathological inflammation or restore anti-tumor immunity, depending on the disease context.

Crucially, iNOS modulation provides a drug-accessible switch that integrates immune function, metabolic reprogramming, and redox signaling. Pharmacological regulation of iNOS—whether through direct enzyme inhibition or upstream transcriptional regulation—can redirect macrophage polarization, offering a novel axis for therapeutic intervention by reconfiguration of macrophage landscape toward homeostatic or disease-resolving phenotypes [27,28].

Macrophage polarization is not a binary process, but rather exists along a functional spectrum, particularly among M2 phenotypes. M2 macrophages are subclassified into M2a, M2b, M2c, and M2d subtypes, based on activation signals, surface marker expression, cytokine profiles, and biological roles. In contrast, M1 macrophages are more uniformly defined by pro-inflammatory activity, with limited recognized subtypes, although functional heterogeneity may exist based on stimulus duration and metabolic programming [40,41].

M2a macrophages are induced by IL-4 or IL-13 and express CD206, Arg1, and Ym1, secreting anti-inflammatory cytokines such as IL-10 and TGF-β. These cells are primarily involved in tissue repair, fibrosis, and defense against parasites [42]. M2b macrophages arise in response to immune complexes in combination with LPS or IL-1β, and are unique in their mixed cytokine profile, producing both IL-10 (anti-inflammatory) and pro-inflammatory cytokines like TNF-α and IL-1β. Functionally, they are associated with immune regulation and Th2 skewing [43]. M2c macrophages are driven by IL-10, TGF-β, or glucocorticoids, and are marked by expression of CD163 and CD206. These cells are implicated in matrix remodeling, immunosuppression, and resolution of inflammation [44]. M2d macrophages, often referred to as tumor-associated macrophages (TAMs), are induced by TLR ligands in the presence of adenosine or IL-6, and secrete VEGF and IL-10. They play crucial roles in angiogenesis, tumor progression, and immune evasion within the tumor microenvironment [45,46,47].

**Figure 2:**
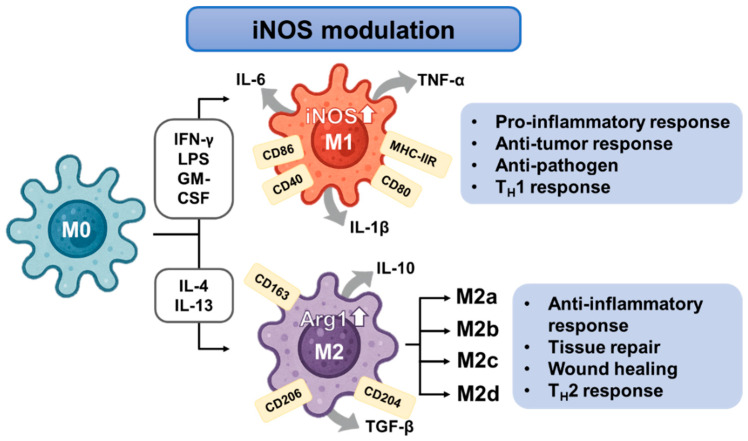
Schematic representation of macrophage polarization and iNOS modulation.

Together, these classifications highlight the plasticity of macrophages and underscore the importance of contextual cues in determining functional phenotype, with therapeutic implications for modulating macrophage responses in disease-specific settings (Figure 2).

## 3. Synthetic Compounds Influencing iNOS Expression and Macrophage Polarization

Inducible nitric oxide synthase (iNOS) plays a pivotal role in macrophage polarization, particularly in promoting the M1 (pro-inflammatory) phenotype via nitric oxide (NO) production. Synthetic iNOS inhibitors have traditionally been explored as anti-inflammatory compounds, particularly in diseases driven by excessive M1 polarization. However, recent findings highlight the dual nature of iNOS regulation, wherein selective inhibition may unexpectedly augment M1 responses in specific conditions, but alternatively diminish iNOS expression and drive M2 polarization in different contexts.

This duality stems from context-dependent effects and the interaction between NO levels, immune signaling, and metabolic pathways.

In the following, we provide a detailed synthetic iNOS regulators categorized based on their mode of action in macrophage polarization: those that directly suppress iNOS enzymatic activity or expression (e.g., L-NIL, CM292, CM544, 1400 W), and those that indirectly modulate iNOS through upstream signaling pathways such as TLR or metabolic regulators, thereby shaping M1/M2 polarization (e.g., CpG ODN, PBI1).

### 3.1. Direct Synthetic Regulators of iNOS: Enzymatic Activity Inhibition

L-N^6^-(1-iminoethyl)-lysine (L-NIL) is a potent and selective inhibitor of inducible nitric oxide synthase (iNOS), demonstrating approximately 28-fold greater selectivity for iNOS over constitutive isoforms, thereby distinguishing it from less selective analogs such as L-NIO or DL-homoNIL [48]. According to Lu et al., L-NIL profoundly influences immune regulation by modulating macrophage polarization: inhibition of iNOS relieves nitric oxide-mediated nitration of IRF5, thereby enhancing M1 polarization with upregulated expression of IL-12, IL-6, CXCL9, and CXCL10, along with increased MHC II surface expression and augmented CD4^+^ T cell activation [49]. These cellular findings were further validated in vivo using an endotoxin shock model, in which pharmacological inhibition with L-NIL or genetic deletion of iNOS resulted in excessive M1-driven inflammatory responses and increased mortality upon LPS challenge. Collectively, these results demonstrate that L-NIL–mediated inhibition of iNOS exacerbates M1-associated inflammation, thereby uncovering a dual role of iNOS, not only as a marker of M1 macrophages but also as a key regulator that promotes their dedifferentiation to restrain excessive immune activation.

This review further highlights the inflammatory activation of microglia and the immunomodulatory effects of iNOS inhibition. Although microglia and peripheral macrophages originate from distinct developmental lineages, both belong to the broader myeloid family [50]. Therefore, assessing their activation states using macrophage polarization models—particularly those linked to iNOS-mediated pro-inflammatory phenotypes—offers important insights into neuroimmune dynamics within the central nervous system (CNS) [51,52].

CM544 and CM292 are acetamidine-based synthetic compounds developed as selective inhibitors of inducible nitric oxide synthase (iNOS). These molecules have shown effective inhibition of nitric oxide (NO) production in LPS-stimulated microglial models, without significantly altering iNOS protein expression levels, indicating direct suppression of enzymatic activity rather than transcriptional downregulation. This mechanism has been confirmed in murine BV2 microglia, where both compounds reduced LPS-induced inflammatory and cytotoxic responses in a dose-dependent manner [53,54]. In addition to their role as iNOS inhibitors, CM544 and CM292 have been observed to reverse metabolic reprogramming in activated microglia, restoring oxidative phosphorylation parameters and decreasing glycolysis-associated markers such as pyruvate kinase M2 (PKM2) nuclear translocation. These changes reflect a shift toward a less inflammatory or partially “resting” phenotype.

While these results suggest that CM544 and CM292 may modulate macrophage/microglial activation status, direct evidence for classical M2 polarization—such as upregulation of Arg1, IL-10, or CD206—is currently lacking. Their data are more consistent with attenuation of hyperinflammatory M1-like activation rather than a full phenotypic shift toward M2.

N-(3-(Aminomethyl)benzyl)acetamidine (1400 W) is a slow, tight-binding and highly selective inhibitor of human iNOS, with a K_d_ ≤ 7 nM and over 5000-fold selectivity compared to eNOS. The inhibition is NADPH-dependent and follows saturation kinetics, with a maximal rate constant of 0.028 s^−1^ and a binding constant of 2.0 μM. L-Arginine competes with 1400 W binding (K_s_ = 3.0 μM), indicating that 1400 W targets the arginine-binding site of iNOS. The inhibited enzyme shows no recovery of activity after 2 h, suggesting that 1400 W is either irreversible or extremely slowly reversible [55]. The selective iNOS inhibitor 1400 W has been demonstrated to modulate macrophage polarization in both in vitro and in vivo systems, and has shown particular therapeutic relevance in temporal lobe epilepsy (TLE) models. In mouse hippocampal slice preparations, kainate-induced epileptiform spiking was significantly reduced by 1400 W treatment, suggesting a direct role of iNOS activity in neuronal hyperexcitability. In a rat kainate-induced temporal lobe epilepsy model, short-course administration of 1400 W not only reduced acute epileptiform spiking but also produced long-term disease modification, with marked reductions in spontaneous recurrent seizures, blood–brain barrier disruption, gliosis, and neurodegeneration. Importantly, immunohistochemical analyses revealed a significant decrease in M1-type microglia within hippocampal and limbic regions, consistent with direct iNOS inhibition driving polarization shifts [56].

The selective iNOS inhibitor 1400 W has been evaluated in murine models of renal ischaemia–reperfusion injury, where it demonstrated region-specific immunomodulatory effects.

Pretreatment with the selective iNOS inhibitor 1400 W (10 mg/kg i.p.) preserved renal function and, at 48 h, shifted medullary macrophage polarization toward M1 (Increased CD38, Fpr2, M1/M2 ratio), indicating that direct iNOS inhibition modulates macrophage programs in vivo, albeit with a transient medullary endothelial/inflammatory transcriptional activation that did not translate into chronic dysfunction by 28 days [57].

The selective iNOS inhibitor 1400 W was evaluated in a murine model of renal ischemia–reperfusion injury. Treatment with 1400 W significantly attenuated inflammatory injury and mesenchymal transition in the renal cortex, where both M1 (Fpr2, CD38) and M2 (Arg1, Erg2, c-Myc) marker expression were suppressed. In contrast, in the renal medulla, 1400 W selectively reduced M1 markers while sparing M2-associated genes, thereby dampening pro-inflammatory polarization without compromising reparative responses. These findings highlight the role of direct iNOS inhibition in modulating macrophage polarization in a region-specific manner within the kidney [58].

### 3.2. Indirect Synthetic Regulators of iNOS: Upstream Signaling and Metabolic Regulation

Oligodeoxynucleotides (ODNs) containing cytosine followed by guanine (CpG ODN) motifs are derived from bacterial DNA motifs and stimulate TLR9. CpG have been shown to modulate macrophage functions, particularly by promoting pro-inflammatory activation and enhancing their antitumor potential. In models of pancreatic ductal adenocarcinoma (PDAC), Liu et al. demonstrated that CpG stimulation as a TLR9 agonist induces central carbon metabolic remodeling characterized by enhanced fatty-acid oxidation (FAD) and diversion of tricarboxylic acid (TCA) cycle intermediates for de novo lipid biosynthesis [42].

Interestingly, CpG-activated macrophages exhibit a non-classical polarization state, characterized by the simultaneous upregulation of both iNOS and Arg1. This dual expression pattern deviates from the conventional M1/M2 dichotomy and suggests a distinct activation profile. Collectively, these findings indicate that CpG stimulation reprograms macrophage metabolism in a unique manner—potentially enhancing their ability to overcome CD47-mediated immune evasion and contribute to anti-tumor immunity.

A novel small-molecule TLR4 agonist (pyrimido[5,4-b] indol, PBI1) has been reported to activate macrophages and induce anti-cancer polarization. In breast cancer models, Yao et al. developed an eGFP reporter cell line based on iNOS promoter activity in RAW264.7 cells (RAW:iNos-eGFP) to monitor macrophage polarization in response to tumor-derived signals. Conditioned media from 4T1 or EMT6 cells, both of which represent murine triple-negative breast cancer cells (TNBC), were cultured in 2D/monolayer and 3D/spheroid co-cultures with these cells, indicating suppression of iNOS and M2-like polarization. However, treatment with PBI1 restored iNOS activity and promoted a shift toward the M1 phenotype, indicating increased anti-cancer activity [43]. Synthetic compounds modulating iNOS and their effects on macrophage polarization are summarized in Table 1.

Synthetic iNOS regulators exert bimodal effects on macrophage polarization. While their canonical role lies in suppressing M1 inflammation, selective inhibition may paradoxically promote M1 reprogramming under specific conditions by alleviating iNOS-derived negative regulation. Notably, synthetic iNOS regulators have been widely studied for their M2-promoting and anti-inflammatory effects via suppression of NO production; certain agents such as L-NIL have paradoxically been shown to enhance M1 polarization. This occurs through the relief of NO-mediated negative feedback on pro-inflammatory signaling. A deep understanding of the macrophage–NO–cytokine axis is essential to leverage these molecules for precision immunotherapy and inflammation resolution.

## 4. Natural Compounds Influencing iNOS Expression and Macrophage Polarization

Natural compounds, derived from various plant sources, were shown to have a remarkable capacity to modulate macrophage polarization through diverse signaling pathways, resulting in either suppression or enhancement of iNOS expression. In this section, we categorize natural compounds based on their structural characteristics, discussing representative examples and their immunomodulatory effects on macrophage polarization.

### 4.1. Isoflavonoid

Isoflavonoids such as puerarin have been shown to suppress LPS-induced iNOS expression, NO production, and pro-inflammatory cytokines like TNF-α and IL-1β in macrophages, showing suppressed M1 polarization [61]. Puerarin has been shown to suppress M1 macrophage polarization in both LPS-induced and UUO (Unilateral Ureteral Obstruction)-induced acute kidney injury mouse models, as well as in RAW264.7 macrophages, by disrupting the TLR4/MyD88 signaling axis and inhibiting downstream NF-κB p65 and JNK/FoxO1 activation. This leads to decreased expression of pro-inflammatory mediators—including iNOS, CD86, IL-1β, IL-6, and TNF-α—and contributes to the attenuation of renal inflammation and pathological damage [62].

### 4.2. Saponins

Astragaloside IV (AS-IV) is a tetracyclic triterpenoid saponin derived from Astragalus species. In RAW264.7 macrophages, AS-IV suppressed M1 polarization by reducing iNOS expression and pro-inflammatory cytokines while enhancing arginase-1 and IL-10, thereby promoting M2 features. In renal ischemia–reperfusion injury model, AS-IV reduced renal injury, fibrosis, and macrophage infiltration, accompanied by decreased M1 and increased M2 macrophage populations. Mechanistically, these effects were mediated through inhibition of NF-κB p65/Hif-1α and Hif-1α/Smad7 signaling pathways, indicating that iNOS regulation influences macrophage polarization via upstream indirect targeting rather than direct enzymatic inhibition [63].

Glycyrrhizic acid (GA), a triterpenoid saponin traditionally regarded as an anti-inflammatory agent, has been shown to promote M1 polarization in bone marrow–derived macrophages by upregulating iNOS expression and enhancing nitric oxide (NO) production. This polarization is further supported by increased expression of classical M1 surface markers (CD80, CD86, MHC II) and pro-inflammatory cytokines including TNF-α, IL-12, and IL-6, while a concomitant downregulation of M2-associated markers such as Arg1 and mannose receptor (CD206/MR). These effects enhanced phagocytosis and bacterial killing, and mechanistic analysis showed activation of MAPKs (ERK1/2, JNK, p38) and NF-κB signaling, with JNK and NF-κB p65 nuclear translocation identified as essential upstream drivers of iNOS induction [64].

Dioscin, a steroid saponin, enhanced M1 polarization while suppressing M2 through iNOS regulation in bone marrow-derived macrophages (BMDMs) and monocytic myeloid-derived suppressor cells (MDSCs). Treatment of dioscin increased iNOS, CD86, TNF-α, and IL-6 while reducing Arg1 and CD206 expression. In Azoxymethane (AOM)/Dextran sulfate sodium salt (DSS)-induced colitis-associated colon cancer mice, dioscin reduced tumor burden and shifted macrophages toward an M1 phenotype (increased iNOS, decreased Arg1 expression). These effects were associated with inhibition of NF-κB p65 phosphorylation, indicating indirect upstream modulation of iNOS [65].

### 4.3. Lignan

Arctigenin, a natural lignan derived from Arctium lappa, suppresses inflammation by inhibiting key signaling pathways that drive iNOS expression, including PI3K, AKT, and IKKβ/NF-κB, in LPS-stimulated macrophages. This results in reduced production of pro-inflammatory cytokines (IL-1β, IL-6, TNF-α) and increased expression of anti-inflammatory markers (IL-10, CD204), indicating a shift from M1 to M2 polarization. These effects were dependent on PI3K activity, as they were abolished in PI3K-silenced cells. In vivo, arctigenin attenuated LPS-induced systemic inflammation and 2,4,6-trinitrobenzene sulfonic acid (TNBS)-induced colitis in mice. These findings suggest that arctigenin contributes to macrophage reprogramming by targeting upstream regulators of iNOS, positioning it as a promising therapeutic agent for inflammatory diseases [49].

### 4.4. Polyphenol

Chlorogenic acid (CGA), a natural polyphenol found in coffee and various fruits, has been discovered to possess anti-inflammatory activity and inducible nitric oxide synthase (iNOS) inhibitor [66]. Recent studies suggest that these properties are closely tied to CGA’s capacity to modulate macrophage polarization, shifting macrophages phenotypically.

In glioblastoma, where tumor-associated macrophages (TAMs) are predominantly M2-like due to the immunosuppressive microenvironment. However, CGA reprograms TAMs toward an anti-tumoral M1 phenotype. This effect is mediated by activation of STAT1 and inhibition of STAT6, resulting in increased expression of iNOS, MHC II, and CD11c, and decreased levels of Arg1 and CD206 in glioma–macrophage co-cultures and xenograft models. Notably, CGA-induced M1 macrophages effectively suppressed the growth of glioma (U87) and breast cancer (MFC) cells in co-culture [67]. Lu Wang et al. further demonstrated that CGA promotes M1 polarization in bone marrow-derived macrophages (BMDMs) by upregulating surface markers such as MHC II, CD80, CD86, and CCR7, while downregulating M2 markers like CD206 and Arg1. Additionally, CGA enhanced pro-inflammatory cytokine expression (TNF-α, IL-6, IL-12), bacterial phagocytosis, and killing capacity associated with activation of JAK-STAT1 and NF-κB signaling pathways [68]. CGA has also been shown to regulate macrophage polarization in infectious diseases. In a Klebsiella pneumoniae–induced pneumonia model, CGA promoted M2 polarization of alveolar macrophages (AMs), increased mouse survival, and reduced bacterial burden. Mechanistically, CGA activated SIRT1, which in turn inhibited HMGB1 acetylation and its nuclear-to-cytoplasmic translocation, thereby suppressing inflammatory responses. Silencing SIRT1 or overexpressing HMGB1 reversed these effects, confirming the role of the SIRT1–HMGB1 axis in CGA-mediated macrophage modulation and pneumonia resolution [69].

Collectively, these findings highlight the diverse and context-dependent immunomodulatory effects of natural compounds on macrophage polarization via iNOS regulation, supporting their potential as adaptable immunotherapeutic agents in both inflammatory and tumor settings. The following table summarizes the various natural iNOS regulator compounds and their effects on macrophage polarization (Table 2).

## 5. Challenges and Opportunities in Clinical Translation

### 5.1. Overview of Clinical Translation

One of the foremost translational challenges associated with iNOS inhibition lies in its context-dependent roles across various disease states. As of 2025, a total of 29 clinical trials involving pharmacological iNOS inhibitors have been registered on ClinicalTrials.gov. The most commonly studied reagent is NG-monomethyl-L-arginine (L-NMMA), with 20 trials, followed by GW274150 (4 trials), Nitro-L-arginine methyl ester (L-NAME, 3 trials), 2-Iminobiotin (2-IB, 1 trial), and Shenfu injection (1 trial).

**Figure 3:**
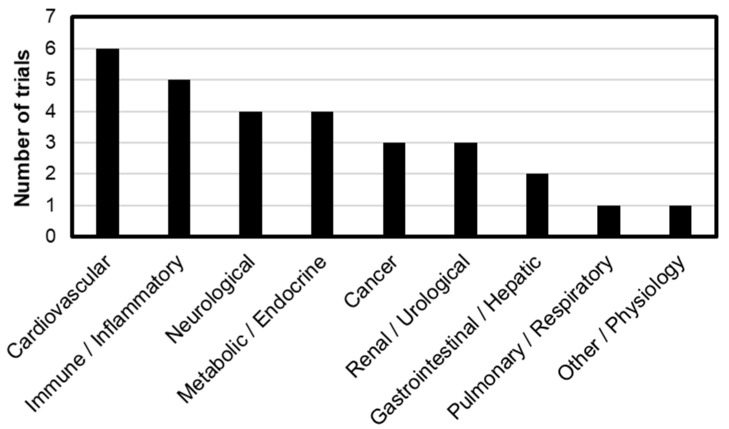
Distribution of 30 clinical trials by primary disease area with number of trials in parentheses: Cardiovascular (6), Immune/Inflammatory (5), Neurological (4), Metabolic/Endocrine (4), Cancer (3), Renal/Urological (3), Gastrointestinal/Hepatic (2), Pulmonary/Respiratory (1), and Other/Physiology (1).

Figure 3 illustrates the distribution of these trials by organ system, categorized by the primary disease targets: Cardiovascular (6 trials), Immune/Inflammatory (5 trials), Neurological (4 trials), Metabolic/Endocrine (4 trials), Cancer (3 trials), Renal/Urological (3 trials), Gastrointestinal/Hepatic (2 trials), Pulmonary/Respiratory (1 trial), and Other/Physiological conditions (1 trial). Notably, L-NMMA has been predominantly evaluated in cardiovascular diseases, accounting for 4 out of the 6 trials. L-NMMA is a well-characterized iNOS inhibitor known to enhance basal vascular tone and attenuate endothelium-dependent vasodilation [71]. This vascular modulation is presumed to underlie its frequent application in cardiovascular-related clinical studies and appears to be expanding into related therapeutic areas. This expansion trend is also observed in other iNOS inhibitors, which are increasingly being investigated across a range of disease contexts (Table 3).

A particularly noteworthy development is the application of iNOS inhibitors in metabolic disorders, especially those related to lipid metabolism. Among L-NMMA trials, three clinical studies have targeted conditions such as obesity and hyperlipidemia. Recent studies highlight the critical interplay between lipid metabolism and inflammation in conditions such as dyslipidemia and atherosclerosis, where altered lipid profiles including modified LDL and reduced HDL activate immune responses that sustain chronic inflammation [72]. Together, these results support the relationship between chronic inflammation and lipid dysregulation. Therefore, research on iNOS inhibitors is expanding from inflammation- and immune-based studies toward lipid metabolism-related metabolic disorders.

Although iNOS inhibitors have been investigated across a wide range of diseases and therapeutic areas, their clinical development remains limited. Among the 29 clinical trials, 31% are currently in Phase 2. When including combined phases such as Phase 1/2 and Phase 2/3, approximately 51.7% of the trials are currently in Phase 2 development, indicating that a significant proportion of iNOS inhibitors have not progressed beyond this stage. Furthermore, no iNOS inhibitors have received FDA approval to date.

One of the major hurdles of clinical translation has been suggested as the absence of validated predictive biomarkers for macrophage polarization or iNOS activity in clinical settings. Without significant molecular biomarkers for prediction, progression, or treatment, real-time response monitoring remains limited [10,34].

Moreover, species-specific differences in iNOS regulation and macrophage biology between humans and rodents raise concerns about the translatability of preclinical data. Because rodent macrophages often exhibit higher iNOS inducibility, potentially overestimating drug efficacy [73,74].

This challenge may partly stem from the disease-context-dependent nature of iNOS activity, rather than being solely related to disease-specific targeting. Such variability complicates clinical translation and underscores the urgent need for further research to better understand iNOS regulation across different pathological conditions.

### 5.2. Critical Evaluation of Key Clinical Trials

To provide insight into more detailed key clinical trials, the following section reviews trial design, cohort characteristics, primary end-point, safety, and the risk–benefit profile of iNOS inhibitors.

Petros et al. studied L-NMMA in a trial designed to inhibit inducible nitric oxide synthase, which produces nitric oxide that contributes to excessive vasodilatation in patients with septic shock [75]. In septic shock patients with hypotension, L-NMMA effectively dose-dependently increased mean arterial pressure (MAP), systemic vascular resistance, pulmonary vascular resistance, and central venous pressure, highlighting its hemodynamic efficacy. However, it was associated with potential risks, including dose-dependent reductions in cardiac output, which may have impaired tissue perfusion.

In sepsis, L-NMMA also elevated systemic vascular resistance and blood pressure [76], but ultimately increased mortality [77].

In triple-negative breast cancer (TNBC), L-NMMA combined with taxane decreased serum nitric oxide (nitrate/nitrite) and induced tumor reduction with an objective response rate (ORR) of 45.8% [78]. Notably, anticancer effect was observed in heavily pretreated cohorts, including metastatic TNBC patients who had received a median of five prior chemotherapy regimens, chemotherapy-refractory locally advanced breast cancer (LABC) patients, and anthracycline-refractory LABC patients. This effect was associated with differences in the tumor immune microenvironment (TME). Non-responders showed increases in markers of M2 macrophages, intratumoral M2 infiltration, and immunosuppressive cytokines, whereas responders showed an increase in neutrophils and a corresponding decrease in protumor N2 neutrophils, suggesting the possibility of reprogramming to antitumor N1 neutrophils via iNOS inhibition. L-NMMA combined with taxane is generally well tolerated, with blood pressure managed with adjunctive amlodipine, and other adverse events consistent with expected chemotherapy toxicities rather than being directly attributable to L-NMMA.

These results suggest that the risk-benefit balance of L-NMMA, between cardiovascular risks and the therapeutic efficacy, including blood pressure control and anticancer effects, needs to be carefully evaluated.

In the case of GW274150, a phase 2 trial conducted for migraine prophylaxis was generally well tolerated but did not show a significant primary efficacy end-point compared to the placebo, defined as the probability of a migraine headache day [79]. Furthermore, although GW274150 produced a clinically meaningful reduction in exhaled breath nitric oxide (FENO) levels in asthma patients, it failed to improve early (EAR) and late (LAR) asthmatic responses to allergens or airway hyperresponsiveness (Methacholine reactivity) [80]. Singh et al. discussed that the NO/iNOS pathway may not represent the central mechanism governing airway inflammation or reactivity. Instead, multiple alternative pathways, such as peroxynitrite formation, S-nitrosothiol signaling, PGE_2_ regulation, and superoxide-driven oxidative stress, appear to contribute, resulting in selective iNOS inhibition insufficient to yield therapeutic benefits in asthma. These results indicate that selective inhibition of the iNOS pathway alone is insufficient to modulate the key pathophysiological mechanisms of the disease. In both migraine prophylaxis and asthma clinical trials, the GW274150 was well tolerated overall without serious adverse events, demonstrating a favorable safety profile, along with iNOS inhibition. However, the clinical efficacy fell short of expectations, which is a key limitation. Therefore, considering the risk-benefit of GW274150, the drug itself is safe, but therapeutic strategies targeting iNOS do not provide the expected clinical benefit in indications such as migraine. In addition, the difficulty in achieving sufficient therapeutic effect with selective iNOS inhibition alone in pathophysiologically complex diseases is evaluated as limiting its development value.

Wecht et al. studied that L-NAME administration increased orthostatic mean arterial pressure (MAP) during head-up tilt test (HUT) in patients with chronic spinal cord injury (tetraplegia) [81]. However, MAP decreased at 45° HUT position, indicating that it is unclear whether the administered dose may be high enough to persist in prolonged periods. Furthermore, although not statistically significant, plasma renin decreased and aldosterone increased, suggesting a potential role in reducing renin–angiotensin–aldosterone system (RAAS) dependence. This suggests that in tetraplegic patients, where decentralized sympathetic vascular control increases RAAS dependence, vasodilation control through NOS inhibition may have potential utility in reducing dependence on the RAAS and improving orthostatic hypotension (OH). Although there were no serious adverse events in this clinical trial, the statistical power is still limited due to a small sample of subjects and differences in response caused by heterogeneity of spinal lesion in location and degree among participants. Therefore, larger cohorts are required to assess long-term stability and interpretation, such as ensuring sufficient dosage to increase MAP with HUT maneuver, and confirming the conclusion regarding the RAAS effect.

A synthesis of the clinical trial results of each representative iNOS inhibitor (L-NMMA, GW274150, L-NAME) suggests that the clinical performance of iNOS inhibition strategies varies significantly depending on the multiple physiological factors including disease characteristics, trial design, patient heterogeneity, immune and hemodynamic environment, and compensatory physiological mechanisms.

Furthermore, physiological and immunological differences between species, disease models, and study cohorts may be key variables in the therapeutic efficacy of iNOS modulation.

Diverse mammalian cells, neutrophils, granulocytes, erythrocytes, hepatocytes, cardiac myocytes, dendritic cells, myeloid-derived suppressor cells, foam cells, natural killer cells, endothelial cells, and smooth muscle cells, have been reported to exhibit varying levels of arginase or NOS activity [82]. Species-specific differences in NOS isoform expression, L-arginine transporter expression and affinity, metabolic pathways, and cofactors lead to differences in drug activities [73,74].

In addition, each NOS isoform (eNOS, iNOS, etc.) prefers a different arginine pool, and the turnover (synthesis, consumption, and exchange rates) of these pools has been reported to vary within endothelial cells. This implies that multiple intracellular arginine pools with different turnover characteristics exist, suggesting that the efficacy of competitive iNOS inhibitors may vary depending on cell type, species, and metabolic environment [83]. For example, for GW274150 administration in human asthma patients [80], early (EAR) and late (LAR) asthmatic responses to allergens or methacholine (MCh) responsiveness were not inhibited, contrasting with guinea pigs, in which allergen-induced LAR was significantly inhibited [84].

Singh et al. also observed that NO reduction through selective iNOS inhibition did not reduce epithelial 3-nitrotyrosine (3-NT) expression after allergen challenge, which contrasts with the complete inhibition of nitrotyrosine staining in lung tissue by GW274150 in a carrageenan-induced rat model of acute lung inflammation [85]. Furthermore, increased arginase metabolism and iNOS activity have been reported in sepsis and ischemic-reperfusion injury, suggesting that the efficacy may vary depending on the disease model and pathophysiological environment [86,87].

Together, the differences in experimental results across species physiology, disease models, and cohort characteristics reflect genuine biological variability rather than inconsistencies in the pharmacology of NOS inhibitor. The most limiting factor to the clinical translation of iNOS inhibitors is the limited cohort sizes and the context-dependent variability of iNOS activity, highlighting the need for larger cohorts and multi-layered research strategies encompassing diverse pathophysiological conditions to provide a critical foundation for more clearly defining their clinical potential. Considering this variability is important for rigorously assessing the limitations and translational challenges of current iNOS inhibition strategies, while also contextualizing the current findings within the broader limitations of existing experimental evidence. Furthermore, differences in the tumor immune microenvironment (TME) between responders and non-responders observed in TNBC trials suggest that iNOS inhibition may be linked to macrophage polarization and immune cell function. These insights call for further preclinical and clinical studies to elucidate the immune-metabolic roles of iNOS and improve the precision of iNOS-targeted therapies.

## 6. Conclusions and Perspectives

Modulating iNOS expression and activity has been shown to dynamically influence macrophage polarization, reprogramming their phenotype in response to inflammatory and environmental signals.

Synthetic iNOS inhibitors, such as L-NIL and 1400 W, primarily act via direct enzymatic inhibition of iNOS activity, thereby modulating macrophage polarization and inflammatory responses. This context-dependent role of iNOS in macrophage polarization influences the balance between pro-inflammatory M1 and anti-inflammatory M2 phenotypes through modulation of nitric oxide (NO) production. While many synthetic iNOS inhibitors have traditionally been studied as anti-inflammatory agents that suppress M1 polarization, recent evidence reveals a more complex picture. For example, L-NIL can paradoxically enhance M1 activation by relieving NO-mediated negative feedback on macrophage activation, increasing pro-inflammatory cytokine expression and antigen presentation. In contrast, another inhibitor, 1400 W, attenuates M1-like metabolic and inflammatory profiles, promoting shifts toward M2 polarization, particularly in chronic inflammatory and neurodegenerative contexts.

In addition to these direct enzymatic inhibitors, certain synthetic compounds regulate iNOS expression and macrophage polarization indirectly through upstream signaling pathways. CpG ODNs, acting as TLR9 agonists, have been shown to modulate macrophage metabolism and promote pro-inflammatory M1 polarization in cancer models, enhancing antitumor immunity. Similarly, PBI1, a novel TLR4 agonist, restores iNOS expression suppressed by tumor-derived signals and promotes M1-like macrophage reprogramming, increasing anti-cancer activity. These indirect regulators highlight the complexity of iNOS regulation via immune signaling pathways, further emphasizing the importance of cellular context in determining macrophage phenotype and function.

Compared to synthetic inhibitors, natural compounds cannot be strictly categorized as M1- or M2-polarizing agents, since their effects are highly context-dependent, varying with the immune landscape, disease model, dosage, and timing of administration. This context-dependent behavior reflects the plasticity of macrophages, modulated not only by compound structure but also by cellular microenvironment, cytokine milieu, and disease-specific signaling pathways. Actually, signal axes such as STAT3, JAK/STAT, NF-κB, and cross-talk among these pathways dynamically orchestrate macrophage phenotype in response to external stimuli [88,89]. These mechanistic differences highlight the importance of evaluating each natural compound independently across different disease contexts, rather than simply classifying them as anti-inflammatory or anti-cancer agents. Side-by-side comparative studies using the same compound in both inflammatory and tumor microenvironments would be essential to identify molecular switches that determine whether a compound promotes or suppresses M2 polarization.

This dual modulatory capacity embodies the concept of immunological plasticity, showcasing the adaptability of immunoregulators across diverse pathophysiological conditions. However, this versatility introduces challenges for therapeutic targeting, as the same compound may switch macrophage polarization states depending on the context.

Therefore, systematic and comparative research applying identical iNOS inhibitors in both inflammatory and oncologic models is critical. Clarifying this dual functionality is essential for safely harnessing iNOS inhibitors as immunomodulatory agents and avoiding unintended consequences, such as tumor-supportive M2 activation during cancer therapy.

Moreover, a deeper understanding of macrophage plasticity will be key to developing targeted therapies that leverage innate immunity for more effective and personalized treatment of inflammatory disorders. Advances in single-cell atlasing and transcriptomic profiling of macrophages now enable precise identification of iNOS-expressing subpopulations, allowing dynamic monitoring of polarization states and paving the way for precision-targeted iNOS modulation in disease-specific contexts [90].

In this context, immunometabolic approaches such as single-cell sequencing can be used to characterize iNOS^+^ macrophage subpopulations across diverse pathological conditions, define their disease-specific metabolic programs, and identify subsets selectively expanded during chronic inflammation or tumor progression. These insights may accelerate the development of iNOS-based macrophage modulation strategies tailored to specific pathological microenvironments.

Modulating macrophage function through iNOS expression and metabolic pathways offers a promising complementary approach to reprogram immune responses. Interestingly, recent studies have suggested that lipid metabolism plays a key regulatory role in macrophage-mediated anti-tumor activity [4,59,91].

Furthermore, clinical trials investigating iNOS inhibitors have revealed significant associations between iNOS modulation and lipid accumulation in macrophages, highlighting an emerging research direction at the intersection of macrophage biology, inflammatory control, and lipid metabolism.

Based on these observations, combining iNOS inhibitors with metabolic modulators—such as agents targeting fatty acid oxidation or amino acid metabolism—may provide synergistic therapeutic benefits by reshaping macrophage immunometabolism toward disease-specific functional phenotypes.

Moving forward, effective macrophage-targeted immunotherapy will require the integration of high-resolution phenotyping, metabolic profiling, and molecular intervention. By uniting high-dimensional macrophage atlasing with targeted pharmaco-logical modulation of iNOS, it becomes possible to mechanistically reprogram macrophage states through immunometabolic rewiring. This combined strategy offers substantial potential for developing context-adapted immunotherapies capable of addressing the dynamic and heterogeneous immune microenvironments that define chronic inflammatory diseases and tumor progression.

## Figures and Tables

**Table 1:** Summary of synthetic compounds affecting iNOS expression via modulation of macrophage polarization.

No	Synthetic Regulator	iNOS Regulation	Target Disease	Polarization	Outcomes	Ref
1	L-N^6^-(1-iminoethyl)-lysine (L-NIL)	Direct (selective iNOS enzymatic inhibition)	- In vitro: myeloid cell (bone marrow–derived macrophages, CD4^+^ T cell co-culture) - In vivo: endotoxin shock (LPS-induced sepsis model)	M1	- iNOS inhibition reduced IRF5 nitration and increased IL-12, IL-6, CXCL9/10, MHC II, CD4^+^ T cell activation - L-NIL-treated or iNOS^−^/^−^: increased M1 response, mortality after LPS challenge - Administration: L-NIL (2%) in drinking water for 7 or 18 days	[49]
	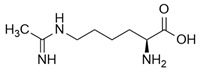					
2	Acetamidine-based iNOS inhibitor (CM544)	Direct (selective iNOS enzymatic inhibition)	In vitro: BV2 microglia, human monocytes	Reduction in M1-like	- In BV2: Reduced NO and ROS production, glycolytic enzyme modulation, increased PKM2 expression - In monocytes: decreased IL-6 release, nitrotyrosine formation, and cell migration, Nrf2 engagement - Administration: CM544 (0–400 μM) treatment	[53]
	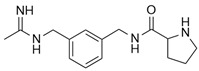					
3	Acetamidine-based iNOS inhibitor (CM292)	Direct (selective iNOS enzymatic inhibition)	BV2 microglia, immortalized microglia from hSOD1(G93A) mice	Reduction in M1-like	Reduced NO production without altering iNOS protein; prevented nitrosative cytotoxicity; restored oxidative phosphorylation, increased OCR and PDH expression, counteracting LPS-induced glycolytic shift - Administration: CM292 (0–200 μM) treatment	[54]
	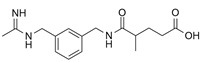					
4	N-(3-(Aminomethyl)benzyl)acetamidine (1400 W)	Direct (selective iNOS enzymatic inhibition)	- In vitro: mouse hippocampal slices (kainate, MEA recording) - In vivo: rat kainate-induced temporal lobe epilepsy model	Reduction in M1	- Reduced epileptiform activity, >90% reduction in spontaneous recurrent seizures in long-term - Suppressed gliosis, 3-NT, M1-type microglia, and neurodegeneration - Administration: In vitro brain slice electrophysiology (5 μM, perfusion); In vivo early epileptogenesis (6 doses q 12 h, 20 mg/kg, i.p.), BBB study (single dose, 20 mg/kg, i.m.)	[56]
			In vivo: renal ischemia–reperfusion injury	M1 promoting	- Renal medulla: upregulation of M1 marker (CD38, Fpr-2), only upregulation M2 marker (Erg-2) → Increase M1/M2 ratio - Administration: single dose (10 mg/kg, i.p.) before reperfusion	[57]
	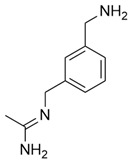			Cortex: Reduction of M1, M2	- Reduced renal damage markers (NGAL, Clusterin), suppressed M1 (Fpr2, CD38) and M2 (Arg1, Erg2, cMyc) markers; IL-6 and TNF-α reduced - Administration: single dose (10 mg/kg, i.p.) before reperfusion I/R injury	[58]
				Medulla: Reduction of M1, M2 preserved	- Suppressed M1 markers, maintained M2 markers, allowing repair programs to persist - Administration: single dose (10 mg/kg, i.p.) before reperfusion I/R injury	
5	CpG oligodeoxynucleotide (CpG ODN)	Indirect upregulator (TLR9 agonist)	Pancreatic ductal adenocarcinoma (PDAC)	Non-classical	Reprograms central carbon metabolism to increase FAO, lipid biosynthesis, iNOS, and Arg1 without affecting classical M1 (MHC-II) or M2 (CD206) markers; induces both pro- (IL-6, IL-12, CCL2, TNF) and anti-inflammatory (IL-10) cytokines - Administration: 100 μg/mL ODN1826 CpG DNA in PBS	[59]
6	Pyrimido [5,4-b] indole (PBI1)	Indirect upregulator (TLR4 agonist)	Breast cancer (4T1 or EMT6 cells represent murine triple-negative breast cancer cells (TNBC))	M1	- TLR4 agonist PBI1 reverses tumor-mediated iNOS suppression; restores M1 polarization; enhances anti-tumor activity - Administration: 20 µg/mL PBI1 in cell culture media (0.4% DMSO) for 24 h prior to analysis	[60]
	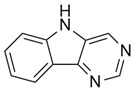					

**Table 2:** Summary of natural compounds affecting iNOS expression via modulation of macrophage polarization.

No	Natural Regulator	iNOS Regulation	Target Disease	Polarization	Outcomes	Ref
1	Puerarin	Indirect (antagonizing TLR4/MyD88,downstream NF-κB p65/JNKFoxO1)	- In vitro: RAW264.7 cells - In vivo: LPS-induced acute kidney injury (AKI) mice, unilateral ureteral obstruction (UUO)-AKI mice	Suppressed M1	- Reduction of iNOS, CD86, IL-1β, IL-6, TNF-α; improved renal pathology and inflammatory status - Administration: In vitro pre-treatment with puerarin (50 µM) for 12 h before inducing polarization; In vivo 3 doses q24h (50 or 100 mg/kg, i.p.), followed by LPS (10 mg/kg, i.p.) on day 4	[62]
	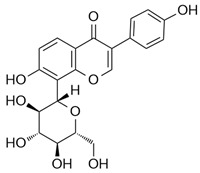					
2	Astragalosides IV (AS-IV)	Indirect (inhibition of NF-κB p65/Hif-1α, Hif-1α/Smad7 signaling)	- In vitro: RAW264.7 cells (LPS + IFN-γ for M1, IL-4/IL-13 for M2) - In vivo: renal ischemia–reperfusion injury model	M1 to M2	- In vitro: Reduction of iNOS, TNF-α/IL-6, increase in Arg-1, IL-10 - In vivo: reduced renal injury, fibrosis, and macrophage infiltration, with decreased M1 and increased M2 macrophages - Administration: In vitro treatment with or without AS-IV under hypoxia (1% O2, 24 h); In vivo 7 doses q24h (20 mg/kg, p.o.) before I/R injury	[63]
	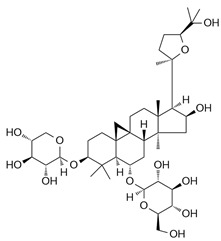					
3	Glycyrrhizic acid (GA)	Indirect (MAPKs-ERK1/2, JNK, p38 and NF-κB p65 nuclear translocation)	- In vitro: mouse BMDMs	Upregulated M1, downregulated M2	- Increased iNOS, increased CD80/CD86, increased TNF-α/IL-6/IL-12; decreased Arg1/MR - Increased phagocytosis and bacterial killing - Administration: GA (100 μg/mL) treatment	[64]
	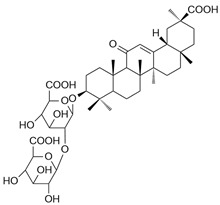					
4	Dioscin	Indirect (inhibition of NF-κB p65 phosphorylation)	- In vitro: BMDMs (LPS + IFN-γ, IL-4); MDSCs - In vivo: AOM/DSS-induced colitis-associated colon cancer mice	Promoted M1, suppressed M2	- In vitro: Increased iNOS, CD86, TNF-α/IL-6, and decreased Arg1 and CD206 - In vivo: Suppressed M2, promoted M1, reduced tumor burden - Administration: In vitro BMDMs treated with LPS (500 ng/mL) or IL-4 (20 ng/mL) ± Dioscin (0.5, 1 μM); In vivo 7doses q48h (2.5, 5, 10 mg/kg dissolved in 0.5% CMC-Na, p.o.)	[65]
	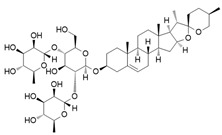					
5	Arctige nin	Indirect downregulator (Inhibition of PI3K/AKT/NF-κB)	- In vitro: LPS-stimulated peritoneal macrophages - In vivo: TNBS-induced colitis mice	M1 to M2	- Inhibition of iNOS, COX-2, NF-κB - Reduction pro-inflammatory cytokines (IL-1β, IL-6, TNF-α) - Increases anti-inflammatory markers (IL-10, CD204) - Administration: In vitro arctigenin (10–20 μM) ± LPS (50 ng/mL) or PGN; In vivo 3 doses q24h (30 or 60 mg/kg dissolved in 2% tween 80, p.o.)	[70]
	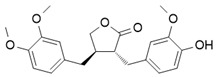					
6	Chlorogenic acid (CGA)	Indirect (activation of NF-κB and JAK-STAT1)	- In vitro: U87 or MFC cells co-cultured with RAW264.7 or Ana-1 macrophages - In vivo: mouse G422 glioma cell xenograft model	M2 to M1	- Increased STAT1 and reduced STAT6 - Elevated iNOS, CD11c, MHC II - Reduced Arg1, CD206 - Inhibit growth of U87 glioma and MFC breast cancer cells - Administration: In vitro CGA (1 or 5 μM) ± M1 induction (LPS 10 ng/mL + IFN-γ 20 ng/mL) or M2 inducer (20 ng/mL, IL-4) for 48 h; In vivo 14 doses q24h (20 or 40 mg/kg, i.p.)	[67]
		Indirect (JAK-STAT1 and NF-κB pathways)	Bone marrow-derived macrophages (BMDMs)	Promoted M1, suppressed M2	- Enhances M1 surface markers and cytokines (CD80, CD86, TNF-α, IL-6); activates JAK-STAT1 and NF-κB pathways - Administration: CGA (50 μg/mL) treatment with IFN-γ (15 ng/mL) + LPS (15 ng/mL) (M1 positive control group), IL-4 (20 ng/mL) (M2 positive control group) for 48 h	[68]
		Indirect (SIRT1 activation → HMGB1 acetylation and nuclear translocation)	K. pneumoniae-induced pneumonia (alveolar macrophages, AMs)	M2 promoting	- Activation of SIRT1 reduces HMGB1 acetylation, thereby suppressing inflammation and enhancing survival - Administration: CGA (200 μM) treatment	[69]
	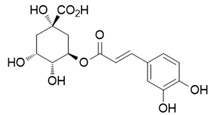					

**Table 3:** Representative iNOS inhibitor in clinical trials (ClinicalTrials.gov).

No	Drug	NCT Number	Condition (Disease)	Phase	Outcomes
1	NG-monomethyl-L-arginine (L-NMMA)	NCT01791816	Vasovagal syncope (VVS) and postural tachycardia syndrome (POTS)	Early phase 1	- Investigates the role of nitric oxide in orthostatic intolerance, including VVS (acute form) and POTS (chronic form) - Hypothesized that enhanced NO reduces sympathetic noradrenergic neurotransmission, leading to splanchnic pooling. NOS inhibitor is used to assess reversal of this mechanism.
		NCT05660083	- HER2-negative breast cancer - Metastatic breast cancer - Metaplastic breast carcinoma	Phase 2	- Advanced metaplastic breast cancer using iNOS inhibitor (L-NMMA), PI3K inhibitor (alpelisib), and chemotherapy (nab-paclitaxel) - Aims to overcome resistance linked to iNOS/NO and PI3K/Akt pathway activation
		NCT03534661	Hyperlipidemias	Phase 3b	- Role of a Nitric Oxide Synthase Inhibitor on GLP-2 Mediated Intestinal Lipoprotein Release - Blocking gut blood flow with L-NMMA is able to prevent GLP-2 from releasing gut lipid stores treated with a combination of Teduglutide
	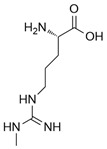				
2	Nitro-L-arginine methyl ester (L-NAME)	NCT00237770	- Hypotension - Spinal cord injury	Phase 2/3	Determine the blood pressure response to NOS inhibition, with L-NAME, in persons with tetraplegia compared to non-SCI control subjects and to establish if blood pressure can be increased while upright in those with tetraplegia
		NCT00835224	- Orthostatic hypotension - Spinal cord injury	Phase 2	Study evaluating the safety and efficacy of L-NAME (NOS inhibitor) and midodrine (alpha receptor agonist) in raising mean arterial pressure (MAP) in persons with chronic tetraplegia
	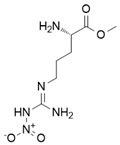				
3	GW274150	NCT00242866	Migraine disorders	Phase 2	- Reduction of NOS, and therefore NO using iNOS inhibitor GW274150 for migraine treatment - Offers anti-inflammatory activity
		NCT00370435	Rheumatoid arthritis	Phase 2	- Evaluation of safety, tolerability and pharmacokinetics - Conducted in adult (over 50 years) and elderly RA patient population on methotrexate
	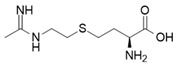				
	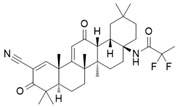				
4	2-Imino biotin (2-IB)	NCT02836340	- Cardiac arrest - Hypoxic–ischemic brain injury	Phase 2	Evaluation of safety, tolerability, and pharmacokinetics of 2-IB administered IV after out-of-hospital cardiac arrest
	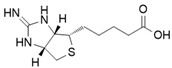				
5	Shenfu injection	NCT03749525	- Septic shock - Vascular reactivity	Phase 2/3	A study investigating the effect of Shenfu injection (traditional Chinese medicine) on improving arterial vascular reactivity in septic shock patients, addressing vasospasm and vascular dysfunction.

## Data Availability

No new data were created or analyzed in this study. Data sharing is not applicable to this article.
